# Association Between Sleep Duration and Depression-Related Symptoms in U.S. Adolescents (2013-2023)

**DOI:** 10.7759/cureus.97236

**Published:** 2025-11-19

**Authors:** Raiyan Faisal, Joan Kimani

**Affiliations:** 1 Research, American International School Dhaka, Dhaka, BGD; 2 Psychology, American International School Dhaka, Dhaka, BGD

**Keywords:** adolescent, adolescent sleep, depression, depression prevention, mental health, risk prediction, secondary data analysis, sleep deprivation, sleep duration

## Abstract

Adolescent sleep deprivation has become a growing public health concern, with recent national surveys reporting sharp declines in sleep duration alongside rising rates of depressive symptoms. This study investigates the correlation between average sleep duration and depression prevalence among U.S. high school students using 10 years (2013-2023) of data from the Centers for Disease Control and Prevention (CDC) Youth Risk Behavior Surveillance System (YRBSS). A Pearson correlation analysis revealed a strong positive association between inadequate sleep and depressive symptoms (r = 0.9010, p = 0.014), with statistical significance defined as p < 0.05. These findings indicate that shorter average sleep duration is significantly associated with a higher prevalence of depressive symptoms among adolescents. While the results do not imply causation, they underscore the importance of monitoring adolescent sleep patterns as a potential correlational indicator of mental health risk in youth populations.

## Introduction

Adolescence is a developmental period marked by heightened vulnerability to mood disorders, yet many young people delay seeking professional help due to stigma or fear of being dismissed. Consequently, early psychiatric symptoms often remain undiagnosed until they progress into chronic [1,2]. Globally, depression affects an estimated 13-15% of adolescents, making it one of the leading causes of disability among youth. In the United States, rates of persistent sadness or hopelessness (PSH) among high school students increased from 28% in 2013 to 42% in 2021, according to the CDC [3-5]. Simultaneously, sleep deprivation affects over 70% of U.S. adolescents. These trends underscore the urgent need to investigate the relationship between insufficient sleep and deteriorating mental health across populations.

Sleep plays a crucial neurobiological role in emotional regulation, memory consolidation, and executive functioning. Both rapid eye movement (REM) sleep, which supports emotional processing, and non-rapid eye movement (NREM) sleep, which facilitates physical recovery, are vital for cognitive and affective stability [4]. Disruption of these stages impairs prefrontal cortex regulation of the amygdala, leading to heightened emotional reactivity and depressive symptomatology [6,7]. Despite mounting evidence linking sleep deficiency to affective dysregulation, few large-scale studies have examined national trends linking adolescent sleep duration and depressive symptoms over time.

Given this gap, the present study explores whether self-reported sleep duration can serve as a potential non-invasive early indicator of depressive risk. Using nationally representative CDC data from 2013 to 2023, this paper examines the long-term correlation between inadequate sleep and the prevalence of depressive symptoms among U.S. adolescents.

We hypothesize that shorter average sleep duration is significantly associated with a higher prevalence of depressive symptoms among U.S. adolescents.

## Materials and methods

Data on adolescent sleep and mental health were obtained from the U.S. Centers for Disease Control and Prevention (CDC) Youth Risk Behavior Surveillance System (YRBSS), spanning 2013-2023 [8]. Datasets were extracted directly from the CDC’s open-access YRBSS repository using the Youth Online Data Analysis Tool, which generates weighted national prevalence estimates for specific indicators across survey cycles. Raw data files were exported in CSV format and compiled using Microsoft Excel (v.16.84, Microsoft® Corp., Redmond, WA). Data visualization and correlation analyses were conducted in Google Sheets (Google, Inc., Mountain View, CA).

The dataset included two variables for each biennial cycle: (1) the percentage of adolescents reporting adequate sleep (>8 hours per night) and (2) the percentage reporting PSH.

Sleep duration

Inadequate sleep duration was quantified from YRBSS self-reported responses to: “On an average school night, how many hours of sleep do you get?” Consistent with CDC and pediatric guidelines recommending eight to 10 hours of sleep per night for adolescents, respondents reporting <8 hours were classified as experiencing inadequate sleep duration [8].

Depressive symptoms (PSH)

Adolescent mental health was assessed using the YRBSS PSH question: “During the past 12 months, did you ever feel so sad or hopeless almost every day for two weeks or more in a row that you stopped doing some usual activities?” This measure incorporates two psychometric qualifiers, chronicity (“almost every day for two weeks or more”) and functional impairment (“stopped doing some usual activities”), which enhance its validity as a proxy for depressive symptoms rather than transient mood states [3,8].

Sampling design

The YRBSS employs a three-stage cluster sampling design of schools, classes, and students to ensure national representativeness of U.S. high school students (grades 9-12, ages 14-18). Both the sleep and PSH variables are derived from the same nationally weighted sample within each survey year, minimizing demographic bias. Each cycle surveyed approximately 14,000-16,000 students, and CDC weighting procedures adjust for age, sex, race/ethnicity, and nonresponse to produce population-level estimates.

Confounder control

Analyses were restricted to national-level, weighted prevalence estimates to reduce demographic confounding. Additional sensitivity checks were conducted by comparing subgroup trends (e.g., sex, grade level, and school type) to confirm the robustness of the observed sleep-mental health association across demographic strata [8].

Ethical considerations

As the YRBSS is an anonymized, publicly available dataset collected by the CDC, Institutional Review Board (IRB) approval was not required for this study. All data usage complied with the CDC’s public data access and citation guidelines.

## Results

To examine the relationship between adolescent sleep duration and the prevalence of diagnosable depression, time-series analysis was conducted on national public health and sleep survey data spanning the period from 2013 to 2023 (Table 1). This analysis aimed to determine whether declining sleep patterns corresponded with increasing depressive symptoms among U.S. high school adolescents. A clear opposing trend was observed over the 10 years. As shown in Table 1, the percentage of adolescents reporting insufficient sleep increased from 68% in 2013 to 77% in 2023. Meanwhile, the percentage of adolescents with depressive symptoms ranges from 30% to 40%.

**Table 1:** Trends in adolescent sleep and prevalence of depressive symptoms, 2013-2023 [8] Values are expressed as percentages (%). “Inadequate sleep” is defined as <8 hours per night. “Depression-related symptoms” refers to adolescents reporting persistent sadness or hopelessness for ≥2 weeks with functional impairment. Each survey year represents a nationally weighted sample of approximately 13,000–17,000 U.S. high school students (grades 9-12) from the CDC Youth Risk Behavior Surveillance System (YRBSS). Notes: Sample sizes (N) per year are included in the dataset. Statistical significance of temporal trends was assessed using Pearson correlation and t-tests; correlation coefficient r = 0.9010, p = 0.014. Confidence intervals for weighted prevalence estimates are available in the YRBSS dataset. Statistical significance assessed using Pearson correlation and t-test, r = 0.9010, p = 0.014. Statistical significance was considered at p < 0.05.

Year	% Adolescents Reporting Inadequate Sleep (>8 hours)	% Adolescents With Depressive Symptoms
2013	68%	30%
2015	68%	33%
2017	73%	32%
2019	74%	37%
2021	78%	42%
2023	77%	40%

**Figure 1:**
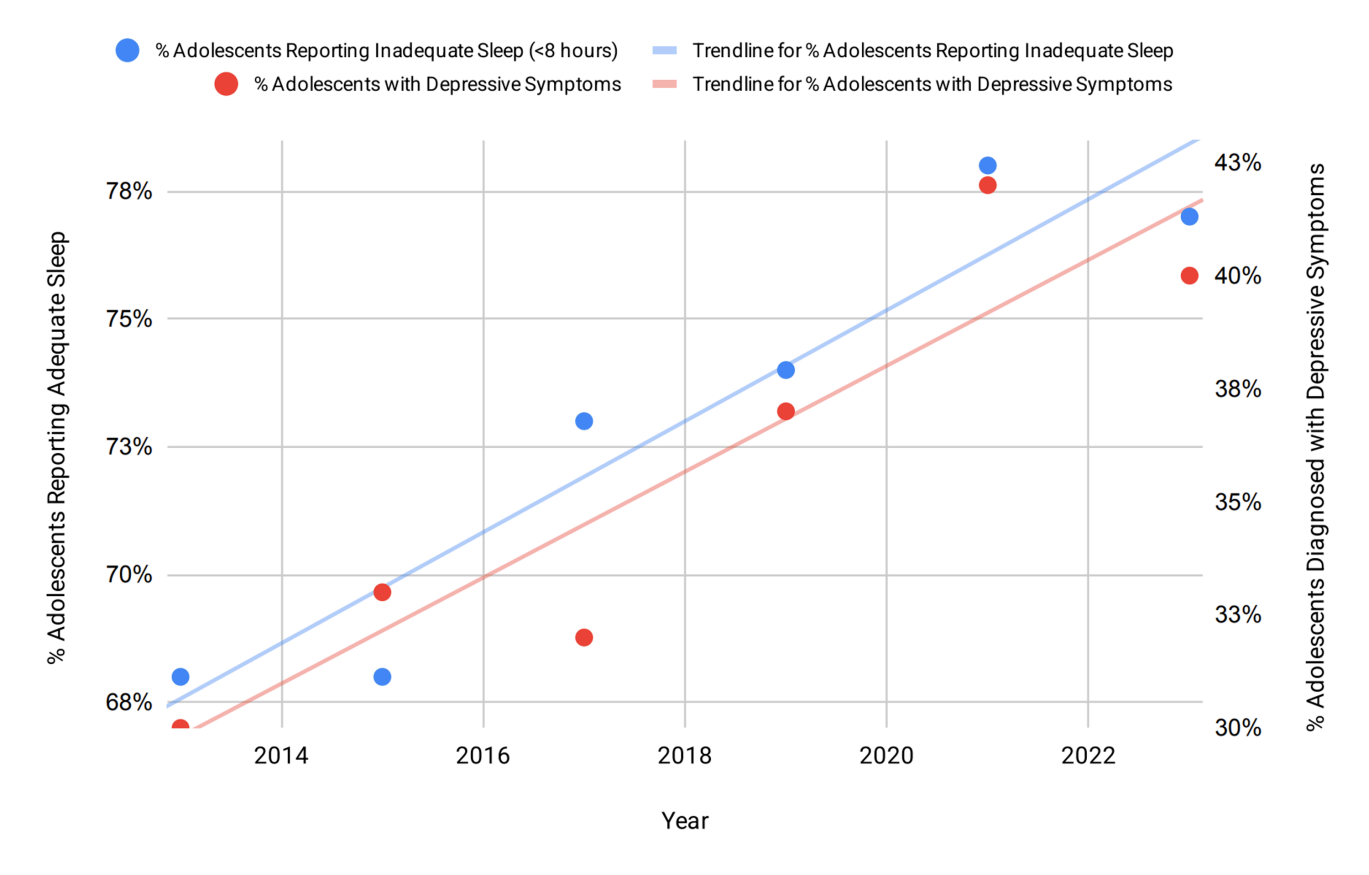
Trends in self-reported adequate sleep and prevalence of diagnosable depression among adolescents (2013-2023). Data show an upward positive trend in sleep alongside a rising trend in depressive symptoms in adolescents. Data show percentages of adolescents reporting inadequate sleep and depression-related symptoms for each survey year. Samples are nationally weighted, ~13,000-17,000 students per year. Data are from the CDC YRBSS. Notes: Pearson correlation and t-tests were used to assess temporal trends; r = 0.9010, p = 0.014. Statistical interpretation is provided in the Discussion section.

The relationship between adolescents reporting inadequate sleep duration and diagnosable depression can further be visualized using a line graph. Figure 1 illustrates two upward trends, suggesting a possible positive association between the two variables. To determine the strength of this relationship, a Pearson correlation coefficient was calculated, yielding r = 0.9010, indicating a very strong positive correlation between inadequate sleep duration and diagnosable depression prevalence. To assess whether this correlation was statistically significant, a t-test for the Pearson correlation was conducted. The test yielded t(4) = 4.15, p = 0.014, confirming that the observed association is statistically significant and unlikely to have occurred by chance, thereby supporting the hypothesis that higher rates of inadequate sleep are associated with increased prevalence of diagnosable depression among adolescents.

## Discussion

The study demonstrates a robust association between inadequate sleep duration and depression-related symptoms among U.S. adolescents. These findings support the hypothesis that increasing rates of insufficient sleep are linked to a higher prevalence of depressive symptomatology. Biological and circadian changes during adolescence likely contribute to this pattern. As teens mature, natural sleep windows shift later, and when combined with early school start times and rising academic or social demands, chronic sleep restriction can occur [5]. Prolonged insufficient sleep disrupts prefrontal cortex activity, impairing emotional regulation and decision-making, while simultaneously increasing amygdala hyperreactivity, the brain region responsible for fear and stress responses [4]. These neurological mechanisms are consistent with patterns observed in depressive symptomatology, hinting at biological plausibility to the observed association.

The findings also highlight the potential utility of sleep duration as a non-invasive indicator of mental health risk. While this study does not establish causality, the strong correlation (r = 0.9010) and consistency across a decade of nationally representative data suggest that monitoring adolescent sleep could help identify individuals at risk of developing depression-related symptoms. Future research could build on these results through longitudinal studies or interventional designs to test whether improving sleep duration can mitigate depressive outcomes.

Study limitations

However, while these findings highlight a strong association, it is important to note that both sleep duration and mental health outcomes were measured through self-report, which can be affected by recall bias and subjective interpretation. Furthermore, the PSH metric reflects persistent depressive symptoms rather than a formal diagnosis, serving as a proxy rather than a direct clinical measure [8]. Nevertheless, the consistency of trends across a decade and the use of a nationally representative, large-scale dataset strengthen the credibility of these results. These considerations suggest that, while causal inferences cannot be made, the observed relationship between inadequate sleep and depression-related outcomes is both meaningful and robust.

## Conclusions

This study demonstrates a strong association between inadequate sleep duration and increased rates of depression among adolescents. The findings underscore the relevance of sleep behavior as a measurable correlate of adolescent mental health status. Future research should employ cohort or case-control designs to further clarify the directionality and causality of this relationship. Expanding such analyses may enhance understanding of how sleep-related interventions could contribute to the early detection of depressive disorders in youth populations.
